# Supplementary material for: ﻿Honoring the Afro-Colombian musical culture with the naming of Epipedobatescurrulao sp. nov. (Anura, Dendrobatidae), a frog from the Pacific rainforests
Source: Zookeys. 2025 Feb 6;1226:139–70. doi: 10.3897/zookeys.1226.123803 (PMC11826230; doi:10.3897/zookeys.1226.123803)
Supplement: Supplementary material 3 — Lateral, dorsal, and ventral images of the type series of Epipedobatescurrulao [file zookeys-1226-139_article-123803__-s003.pdf]

Epipedobates currulao type series

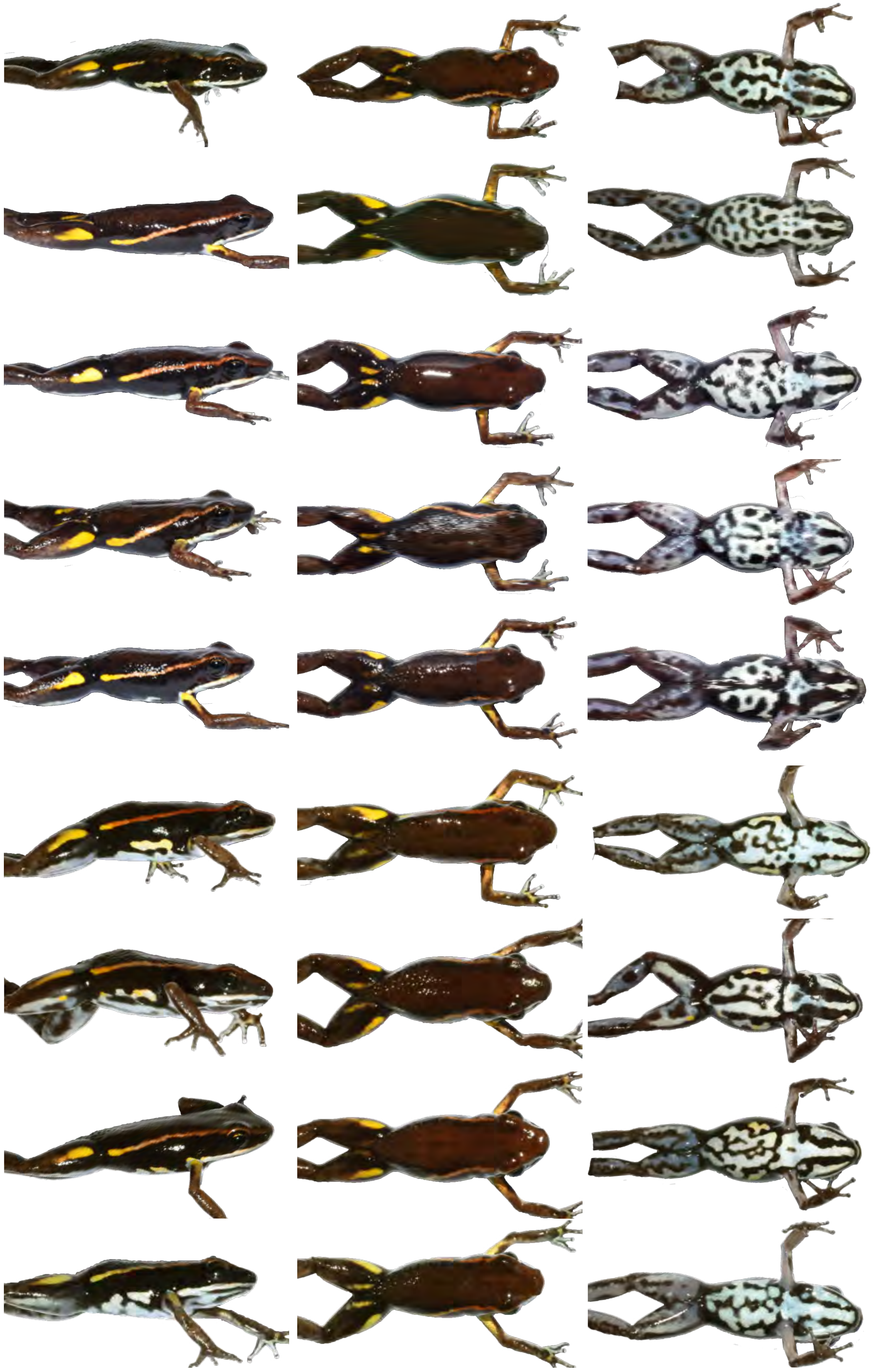

ANDES:A:5256
AJC07731 ♂

ANDES:A:5257
AJC07733 ♀

ANDES:A:5258
AJC07734 ND

ANDES:A:5260
AJC07735 ♂

ANDES:A:5261
AJC07736 ♂

ANDES:A:5262
AJC07737 ♀

ANDES:A:5263
AJC07738 ♂

ANDES:A:5264
AJC07739 ♀

ANDES:A:5265
AJC07741 J

Betancourth-Cundar et al. 2024: Suppl. material 3A

Epipedobates currulao type series

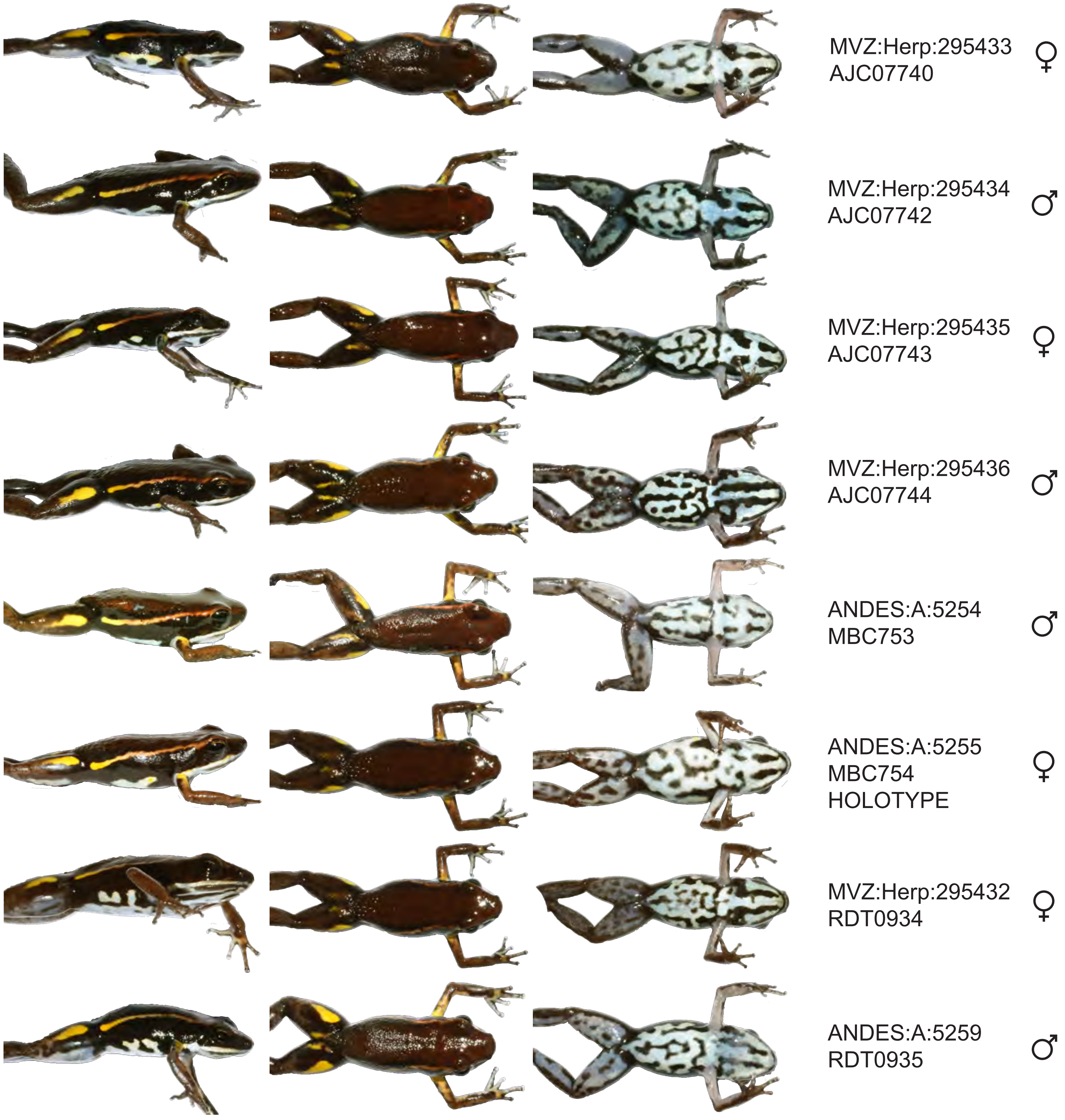

Betancourth-Cundar et al. 2024: Suppl. material 3B
