# Supplementary figures and images for: ﻿Honoring the Afro-Colombian musical culture with the naming of Epipedobatescurrulao sp. nov. (Anura, Dendrobatidae), a frog from the Pacific rainforests
Source: Zookeys. 2025 Feb 6;1226:139–70. doi: 10.3897/zookeys.1226.123803 (PMC11826230; doi:10.3897/zookeys.1226.123803)

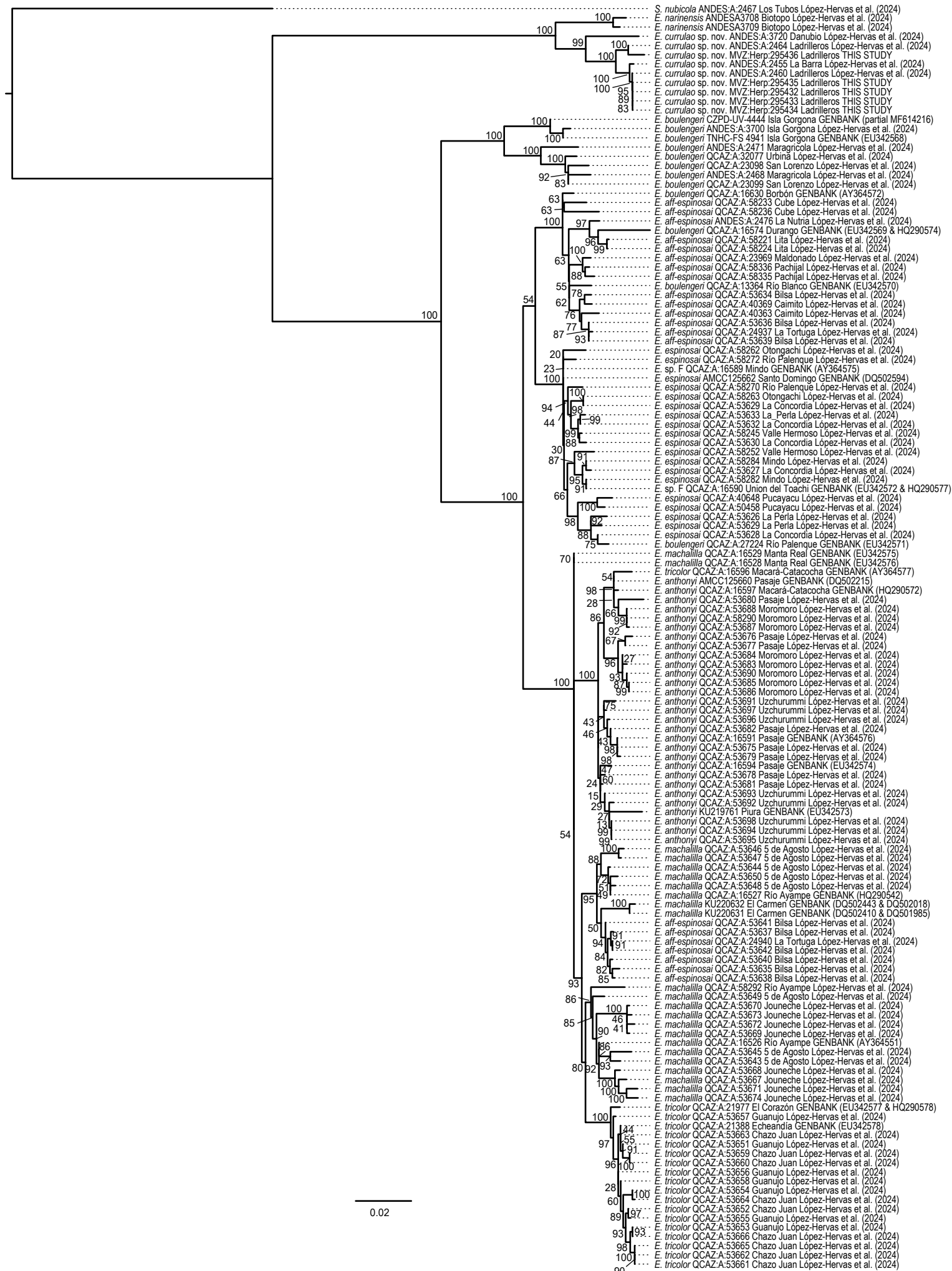

0.02

Supplement: Supplementary material 6 — The optimal likelihood tree obtained from IQ-TREE with bootstrap values and specimen information [file zookeys-1226-139_article-123803__-s006.pdf]
